# The benefit of diet on paradoxical breathing and sleep in Osteogenesis imperfecta

**DOI:** 10.1186/s13023-025-04146-9

**Published:** 2025-11-27

**Authors:** Antonella LoMauro, Ramona De Amicis

**Affiliations:** 1 Dipartimento di Elettronica, Informazione e Bioingegneria, Politecnico di Milano, piazza Leonardo Da Vinci, 20133 Milan, Italy; 2 International Center for the Assessment of Nutritional Status and the Development of Dietary Intervention Strategies (ICANS-DIS), Department of Food, Environmental and Nutritional Sciences (DeFENS), University of Milan, 20133 Milan, Italy; 3 IRCCS Istituto Auxologico Italiano, Obesity Unit and Laboratory of Nutrition and Obesity Research, Department of Endocrine and Metabolic Diseases, Milan, 20145 Italy

## Abstract

We evaluated breathing (i.e.: thoracic contribution to tidal volume in the supine position) and sleep (i.e.: apnea-hypopnea index (AHI)) before and after a 6-month of restricted Mediterranean Diet on 22 volunteers with a confirmed diagnosis of Osteogenesis Imperfecta (median age: 37.8 years; 17 women; 13 type III). At the end of the 6 months, 8 individuals (median age: 38.1 years, 5 females, 3 type III) did not spontaneously follow the diet therefore serving as the control group (CtrOI) for the 14 (median age: 34.7 years, 10 females, 9 type III) who completed the program (DietOI). AHI tended to decrease in DietOI, while it even tended to increase in CtrOI. The thoracic contribution to tidal volume in the supine position of DietOI passed from almost no expansion before diet to a significant expansion after diet; while it was negative in CtrOI indicating systematic paradoxical breathing in the supine position. This pilot study show that the main beneficial effect of diet was a to significantly expand the thorax in supine position with a tendency to reduce the AHI index.

To the editor.

In the last five years, we dedicated our attention to the relationship between three important vital functions in Osteogenesis Imperfecta (OI): nutrition, sleep and breathing. Three papers published in this journal were the results of our research [1–3].

In the first paper, we have shown that the pathophysiology of OI ensues a dangerous vicious circle among breathing, sleep and nutritional status that negatively affects the quality of life. This vicious circle is fed by both intrinsic characteristics of the disease (i.e.: thoracic, cranial and mandibular deformities) and bad daily habits of the patients (i.e. physical inactivity and low dietary quality). The final result of the vicious circle is restricted respiratory function and Olers being more prone to experience overweight or obesity with a high incidence of obstructive sleep apnea (OSA) as the main consequence. We have also identified the main features predisposing OI to OSA: type III form of the disease, neck-to-height ratio of over 31.6%, and paradoxical breathing in the supine position [1].

In the second paper, we have concentrated on the nutritional aspect of the disease, a surprisingly neglected area in the scientific literature. Our patients exhibited compromised dietary quality, characterized by poor adherence to the Mediterranean diet, with limited consumption of whole foods and excessive intake of noxious foods and confectionery. We have therefore conducted a 6-month restricted Mediterranean Diet study on OI patients. The restricted Mediterranean Diet, designed to reduce total daily energy expenditure by 30%, was rich in nutrients crucial for bone health, such as vegetable proteins, vitamins B, D, and E, omega-3 fatty acids, oleic acid, and selenium, calcium, and polyphenolic compounds. The results demonstrated significant improvements in weight and fat mass, observed in all patients at the neck, waist, and limb levels, accompanied by the maintenance of markers of muscle mass and protein stores, energy expenditure, and glucose and lipid profiles in the patients who adhered to the diet [2].

In the third paper, we have discerned the separate impact of the disease and obesity on sleep and respiratory restriction. We have shown that OI alone implies a 30% prevalence of OSA, restricted lung and ribcage, and rapid and shallow breathing in the seated position. The additional impacts of obesity on OI determine a higher incidence of OSA and paradoxical breathing in the supine position together with hypoventilation in the seated position. These results confirmed the importance of reversing obesity in OI to limit the restricted effect on breathing and improve sleep quality [3]. Notably, while a healthy, balanced diet is generally advised for patients with OI, formal and detailed dietary recommendations are often absent in the main management reviews for this condition. This represents a significant gap, especially considering the high prevalence of overweight, obesity, and inadequate nutritional intake reported in this population [4, 5]. However, an emerging body of evidence underscores the positive impact of nutritional interventions. For instance, a study by Zambrano and collaborators demonstrated that a nutritional intervention in pediatric patients with OI significantly improved calcium intake and bone mineral density [6]. Furthermore, the importance of managing BMI is highlighted by findings that link higher body fat percentage with an increased number of fractures in OI patients [5]. Our study, therefore, aims to contribute to this growing evidence base by specifically evaluating the effect of a targeted diet on the often-overlooked respiratory comorbidities.

The last element needed to complete our research was to evaluate the effect of diet on sleep and breathing in OI. We hypothesised that an adequate dietary intake may break or at least ameliorate the vicious circle found in the first paper. The rationale was that reducing excessive adipose tissue located around the neck and the abdomen may have positive mechanical effects on lung and chest wall expansion and work of breathing, oxygen cost of breathing and upper airway obstruction. Before implementing the same multidisciplinary protocol as the first study, which is demanding in terms of organization, resources, time and budget, we ran a smaller pilot study to test our hypothesis. We evaluated breathing and sleep before and after a 6-month restricted Mediterranean Diet on 22 volunteers with a confirmed diagnosis of moderate (type I or IV) or severe (type III) OI (median age: 37.8 years; 17 women; 13 type III). The diet was tailored for each subject according to the experience earned from the second study [2]. We have considered only the most representative outcomes of the first study for sleep and breathing, namely the apnea-hypopnea index (AHI) and the percentage contribution of the pulmonary ribcage to tidal volume in the supine position. The latter serves to quantify the paradoxical breathing in the same position as sleeping and it was measured using opto-electronic plethysmography. The former is the number of apnea and hypopnea events per hour of sleep, indicating the severity of sleep apnea and it was measured with a home portable monitoring for the diagnosis of sleep-disordered breathing (no hospitalization needed for the subjects). At the end of the 6 months, 8 individuals (median age: 38.1 years, 5 females, 3 type III) did not spontaneously follow the diet, therefore serving as the control group (Ctr_OI_) for the 14 (median age: 34.7 years, 10 females, 9 type III) who completed the program (Diet_OI_). By definition, body mass index significantly decreased in Diet_OI_, while it even tended to increase in Ctr_OI_ (*p* = 0.06, left panel of Fig. 1). A similar trend was found for AHI, although it did not reach a statistical difference (middle panel of Fig. 1). The pulmonary ribcage contribution to tidal volume in the supine position of Diet_OI_ passed from almost no expansion before diet (mean value around zero) to a significant expansion after diet (positive contribution values). By contrast, the contribution of the pulmonary ribcage to tidal volume was negative in Ctr_OI_, indicating systematic paradoxical breathing in the supine position (right panel of Fig. 1). There was no impact on Forced Vital Capacity in Diet_OI_ (median pre: 69.7, pre interquartile range: (58.2–76.0) %; post: 66.1 (56.8–74.4) %; *p* = 0.550) and CTR (pre: 70.0 (60.4–76.6) %; post: 67.5 (59.6–80.3) %; *p* = 0.867). Similarly, diet did not impact Total Lung Capacity in Diet_OI_ (pre: 71.5 (41.8–83.0) %; post: 68.0 (45.5–80.0) %; *p* = 0.953) and CTR (pre: 71.0 (55.0–75.0) %; post: 68.0 (58.0-73.8) %; *p* = 0.901).

**Fig. 1:**
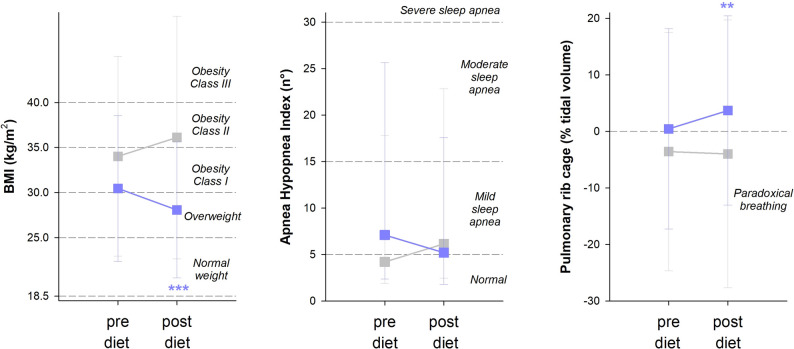
Median (symbols), 25th percentile (lower whiskers) and 75th percentile (upper whiskers) of BMI (computed as weight/height^2^, left panel), AHI index (middle panel) and the percentage contribution of pulmonary ribcage to tidal volume at rest in the supine position (computed with opto-electronic plethysmography, right panel) before (pre-diet) and after (post-diet) 6-month restricted Mediterranean Diet in the individuals who did not spontaneously follow the diet (grey) and in the individuals who completed the program (violet)

Although the protocol was very simple, these data support our hypothesis because the main effect of diet was a significant expansion of the pulmonary ribcage in the supine position with a tendency to reduce the AHI index. We have therefore initiated a virtuous circle to counteract the vicious one that we highlighted in the first study [1]. The improvement of body composition, secondary to the reduced BMI, results in: (1) decreased body weight (43.5 kg vs. 40.4 kg, *p* < 0.001); (2) decreased truncal fat indicated by the reduced neck circumference (36.5 cm vs. 34.5 cm; *p* < 0.001) and (3) decreased abdominal fat indicated by the reduced waist circumference (83.6 cm vs. 79.6 cm; *p* < 0.001) found in Diet_OI_ but not in Ctr_OI_ (weight: 55 kg vs. 55.2 kg, *p* = 0.008; neck: 37.3 vs. 36.1 cm, *p* = 0.321; waist: 90.1 vs. 91.1 cm, *p* = 0.07). Given that fat accumulation around the neck and abdomen imposes a mechanical load on the thorax during respiration, the dietary intervention primarily aimed to alleviate the burden on the respiratory muscles. Notably, the pulmonary ribcage, along with the underlying upper lung lobe, finally expands (although still to a limited extent) only as a result of the diet. Furthermore, as neck fat also creates a mechanical load on the airway during sleep, the diet seems to reduce the strain on throat muscles. Consequently, the AHI demonstrated a trend toward normalization post-intervention.

Despite the limitations of this pilot study (namely, the small sample size and the simplified protocol), the preliminary data strongly suggest the need for further, comprehensive analysis. Future research should adopt a multidisciplinary, international approach to recruit larger and more diverse populations. While this pilot assessed a limited number of parameters, expanding the test battery would be valuable for a more thorough understanding and description of the pathophysiological mechanisms related to body composition, sleep disorders, and respiratory ventilation in OI. International collaboration is essential to compare the efficacy of the Mediterranean diet with other widely adopted dietary patterns, such as the Atlantic, Nordic, American, and Vegetarian diets. Finally, stratifying data by BMI and disease severity is crucial. Our intervention group included 6 normal weight, 3 overweight, 3 obesity class I, 1 obesity class II, and 1 obesity class III. Our control group included 1 normal weight, 3 overweight, 3 obesity class I, 3 obesity class II, and 3 obesity class III.

In conclusion, addressing obesity in OI is essential to mitigate the compounding effects of excess weight on OSA syndrome and supine paradoxical breathing. Our preliminary data contribute to the growing body of evidence that targeted dietary interventions can mitigate not only metabolic risks but also key comorbidities, such as supine paradoxical breathing, in individuals with Osteogenesis Imperfecta. Given that poor nutritional habits, low calcium intake, and a high prevalence of obesity are well-documented in the OI population, and that excess weight is associated with increased morbidity, including a higher fracture risk, the need for proactive nutritional management is clear. We therefore strongly agree that the ultimate goal must be the development of an international consensus on evidence-based dietary recommendations for individuals with OI. Such guidelines are essential to standardize care, improve quality of life, and reduce the overall burden of the disease.

## Data Availability

The data supporting this study’s findings are available upon request from the corresponding author ALM. The data are not publicly available due to restrictions since they contain information that could compromise the privacy of research participants.
